# SER Performance of Enhanced Spatial Multiplexing Codes with ZF/MRC Receiver in Time-Varying Rayleigh Fading Channels

**DOI:** 10.1155/2014/537272

**Published:** 2014-07-10

**Authors:** In-Ho Lee

**Affiliations:** Department of Electrical, Electronic and Control Engineering, Hankyong National University, Anseong 456-749, Republic of Korea

## Abstract

We propose enhanced spatial multiplexing codes (E-SMCs) to enable various encoding rates. The symbol error rate (SER) performance of the E-SMC is investigated when zero-forcing (ZF) and maximal-ratio combining (MRC) techniques are used at a receiver. The proposed E-SMC allows a transmitted symbol to be repeated over time to achieve further diversity gain at the cost of the encoding rate. With the spatial correlation between transmit antennas, SER equations for *M*-ary QAM and PSK constellations are derived by using a moment generating function (MGF) approximation of a signal-to-noise ratio (SNR), based on the assumption of independent zero-forced SNRs. Analytic and simulated results are compared for time-varying and spatially correlated Rayleigh fading channels that are modelled as first-order Markovian channels. Furthermore, we can find an optimal block length for the E-SMC that meets a required SER.

## 1. Introduction

Multiple-input multiple-output (MIMO) schemes are typical for the purposes of maximizing data rate [1] or diversity gain [2, 3]. A maximum data rate is achieved by a spatial multiplexing method [1, 4] by which a single user's data stream is split into multiple substreams and transmits them in parallel using an array of transmit antennas over the same frequency band. A spatial multiplexing code (SMC) given in [1, equation (7.2.1)] is capable of achieving high spectral efficiency. However, it does not achieve spatial transmit diversity since each antenna transmits an independent data stream. Such a SMC has been adopted in cooperative systems as well as cognitive radio systems in order to improve the spectral efficiency [5–7].

In this study, we address enhanced SMCs, referred to as E-SMCs, which extend the conventional SMC in [1, equation (7.2.1)] to transmit the same symbol repeatedly over time and hence enhance temporal transmit diversity instead of losing certain multiplexing gain. In the literature, variable-rate space-time block codes (vr-STBCs) also have been considered as a tool of realizing a tradeoff between the multiplexing and the spatial diversity gain [8, 9]. The vr-STBCs introduce the multiplexing gain into STBCs that achieve the full spatial diversity gain on slow fading channels. Unlike vr-STBCs, E-SMCs achieve time diversity gain on fast varying channels. E-SMCs as well as vr-STBCs are useful for obtaining various encoding rates and unequal error protection in MIMO systems. E-SMCs in this paper use a simple repetition code structure. Since other efficient code structures with coding schemes superior to the repetition coding scheme can improve the performance further, E-SMCs with repetition coding may give a lower bound on the performance.

In decoding E-SMCs, we separate received signals using a zero-forcing (ZF) receiver [4] per time slot and then combine the outputs of the ZF receiver using maximal-ratio combining (MRC) [10] per code block. The MRC is selectively performed only for the repeated symbols in a code block. We use a ZF receiver since it generally has low implementation complexity and makes SER analysis tractable. Furthermore, MRC offers a maximum coding gain of the E-SMC with the ZF receiver.

Given a required symbol error rate (SER) for each data stream, we provide a method that constructs E-SMCs consisting of a minimal number of time slots. And the codes are further optimized to have a maximum encoding rate. We will compare the data rates between E-SMCs and vr-STBCs when required SER is given. E-SMCs perform better than vr-STBCs when the time correlation of radio channels is low since vr-STBCs are designed on the assumption that the channel is constant during a transmitting code block.

To investigate SER performance for various encoding rates of E-SMCs, we assume an equal number of transmit and receiving antennas, an equal power allocation between the transmitters, and perfect channel state information known at the receivers. We also assume that spatial correlation exists only at the transmitters, which is valid when the transmitters are sufficiently high above the local scattering environments [11, 12]. Under these assumptions, SER equations of *M*-ary QAM and PSK constellations are analytically obtained, respectively, by using a moment generating function (MGF) approximation for signal-to-noise ratio (SNR) under the independence assumption on the ZF output SNRs.

With numerical investigation, analytical and simulated results are compared for spatially correlated and time-varying Rayleigh fading channels where the time variability is modelled as first-order Markovian channel [13]. Numerical results show that the analytical SER curve is perfectly matched with the simulated one if the correlation of channels between time slots does not exist, and the gap between the simulated SER result for zero time correlation and that for low time correlation (e.g., less than 0.7 in the numerical results) is negligible, regardless of the spatial correlation. Moreover, the results show that the proposed E-SMCs offer better SER performance, given a data rate, than the conventional SMC at high SNRs. We also study the impact of transmit correlation on the SER performance.

This paper is organized as follows.  Section 2 describes the system and the channel model considered in this paper and introduces the E-SMC.  Section 3 provides approximate SER analysis for E-SMCs with ZF and MRC.  Section 4 presents an optimization of the E-SMC.  Section 5 gives the numerical results in which the analytical results are compared with the simulated ones. Finally, Section 6 concludes the paper.

## 2. System Description

### 2.1. Enhanced Spatial Multiplexing Codes (E-SMCs)

An E-SMC matrix for *N* time slots is defined as the following form:

(1) $$\begin{array}{c} X=\overset{K}{\underset{k=1}{\sum }}x_{k}A_{k}, \end{array}$$

where {*x*_1_,…, *x*_*K*_} is a set of complex symbols to be transmitted and **A**_*k*_ is a fixed code matrix of dimension *n*_*t*_ × *N* for the *k*th complex symbol which satisfies the following properties.

- Each entry of **A**_*k*_ for *k* = 1,2,…, *K* is either 0 or 1.
- The sum of the entries in each column of **A**_*k*_ is either 0 or 1.
- ∑_*k*=1_^*K*^ **A**_*k*_ = Φ, where each entry of *n*_*t*_ × *N* matrix Φ is either 0 or 1.

In the three properties, it is noted that modulo-2 arithmetic is not assumed. Thus, the properties imply that the E-SMC is based on a simple repetition code over time slots and not able to transmit the same symbols over transmit antennas in each time slot. With the E-SMC transmission matrix in (1), *K* complex symbols are transmitted via *n*_*t*_ transmit antennas over *N* time slots, and the encoding rate of an E-SMC is referred to as

(2) $$\begin{array}{c} R_{c}=\frac{K}{N}. \end{array}$$

Two examples of E-SMCs are as follows:

(3) $$\begin{array}{c} X_{1}=\left[\begin{array}{ccc} x_{1,1} & x_{2,2} & x_{3,3}\\ x_{4,1} & x_{1,2} & x_{2,3}\\ x_{3,1} & x_{4,2} & x_{1,3}\\ x_{2,1} & x_{3,2} & x_{4,3} \end{array}\right],\;\;X_{2}=\left[\begin{array}{cc} x_{1,1} & x_{1,2}\\ x_{2,1} & x_{2,2}\\ x_{3,1} & x_{3,2}\\ x_{4,1} & x_{4,2} \end{array}\right], \end{array}$$

where *x*_*k*,*n*_ denotes the *k*th complex symbol at the *n*th time slot for *k* = 1,2,…, *K* and *n* = 1,2,…, *N*. In (3), **X**_1_ and **X**_2_ with *n*_*t*_ = *K* = 4 have *R*_*c*_ = 4/3 and 2, respectively. It is noted that a conventional SMC is a special case of E-SMCs with *K* = *n*_*t*_ and *N* = 1, which is given as ${\left[\begin{array}{cccc} x_{1,1} & x_{2,1} & \cdots & x_{n_{t},1} \end{array}\right]}^{T}$ where [·]^*T*^ denotes the transpose.

### 2.2. Received Signals in Spatially Correlated MIMO Channels

We consider a MIMO system with *n*_*t*_ transmit antennas and *n*_*r*_(≥*n*_*t*_) receiving antennas. Let **X** denote an *n*_*t*_ × *N* transmission matrix, where *N* denotes the code length. Then, the received signal **y**_*n*_ = [*y*_1,*n*_, *y*_2,*n*_,…, *y*_*n*_*r*_,*n*_]^*T*^ is expressed as

(4) $$\begin{array}{c} y_{n}=H\left(n\right)\cdot \sqrt{\frac{P}{n_{t}}}x_{n}+v_{n},\;n=1,2,\ldots ,N, \end{array}$$

where *n*_*t*_ × 1 vector **x**_*n*_ is the *n*th column of transmission matrix **X**, *P* denotes the total average power transmitted on *n*_*t*_ antennas, and **H**(*n*) denotes the *n*_*r*_ × *n*_*t*_ channel matrix at time slot *n*. *n*_*r*_ × 1 vector **v**_*n*_ is the additive white Gaussian noise (AWGN) at a receiver and the AWGN with noise variance *σ* ^2^, given by filtering and sampling the noisy received signal, is modelled by a complex-valued Gaussian vector:

(5) $$\begin{array}{c} v_{n}\sim N_{C}\left(0_{n_{r}\times 1},\sigma^{2}I_{n_{r}\times n_{r}}\right),\;n=1,2,\ldots ,N, \end{array}$$

where 0_*n*_*r*_×1_ is an *n*_*r*_ × 1 column vector with all zero elements and **I**_*n*_*r*_×*n*_*r*__ is an *n*_*r*_ × *n*_*r*_ identity matrix.

We consider time-varying and flat Rayleigh fading channels. We also assume the perfect channel information at a receiver and the presence of only transmit correlation; that is, there is sufficiently rich scattering at a receiver so that the receiver antennas would be uncorrelated. Analogous to a first-order Markovian model used in [13], time-varying and spatially transmit-correlated MIMO channels **H**(*n*) can be modelled as follows:

(6) $$\begin{array}{c} H\left(n\right)=t_{c}\cdot H\left(n-1\right)+H_{w}\cdot R_{t}^{1/2},\;n=1,2,\ldots ,N, \end{array}$$

where *t*_*c*_ is the time correlation factor, **R**_*t*_  (∈*R* ^*n*_*t*_×*n*_*t*_^) denotes the correlation between transmit antennas, and (·)^1/2^ stands for the Hermitian square root of a matrix. Each element of *n*_*r*_ × *n*_*t*_ matrix **H**_*w*_ is an independent and identically distributed (i.i.d.) complex Gaussian random variable with mean zero and variance (1 − *t*_*c*_^2^)/2 per dimension. For the initial channel coefficients, **H**(0) = **H**_0_ · **R**_*t*_^1/2^, where the elements of **H**_0_ are i.i.d. complex Gaussian random variables with mean zero and variance 0.5 per dimension. When *t*_*c*_ = 0, the spatial channel varies independently from one to another time slot. In contrast, for *t*_*c*_ = 1, the channel keeps constant over *N* time slots. Consequently, the flat Rayleigh MIMO channel in this paper is modelled as a time-varying channel with certain correlations during *N* time slots but independent from block to block of the length of *N* time slots. It is noted that the channel is varying very rapidly since we assume that it varies every time slot.

## 3. SER Performance of E-SMCs

### 3.1. SNR of SMCs with a ZF Receiver

When a ZF receiver is used for the received signals in (4), assuming that the receiver has perfect channel information, the ZF output SNR of symbol *k* at time slot *n* is given by [4]

(7) γk,n=Pntσ2[(H(n)∗H(n))−1]ik,nik,n,   k=1,2,…,K, n=1,2,…,N,

where [·]_*ii*_ denotes the (*i*, *i*)th element of a matrix, *i*_*k*,*n*_ is the index of the transmit antenna that is used to transmit symbol *k* at time slot *n*, and ∗ denotes the Hermitian.

Considering that *n*_*t*_ = *n*_*r*_ in order to simplify the statistical analysis, the PDF of *γ*_*k*,*n*_ is obtained by [4]

(8) $$\begin{array}{c} f_{\gamma_{k,n}}\left(\alpha_{k,n}\right)=\frac{1}{\rho c_{k,n}}\exp\left(-\frac{1}{\rho c_{k,n}}\alpha_{k,n}\right),\;\alpha_{k,n}\geq 0, \end{array}$$

where *ρ* = *P*/*σ* ^2^ and *c*_*k*,*n*_ = (*n*_*t*_[**R**_*t*_^−1^]_*i*_*k*,*n*_*i*_*k*,*n*__)^−1^.

### 3.2. MRC Technique for ZF Output Signals

We consider a MRC technique which renders a received SNR maximum. The ZF output signals are combined linearly with the following MRC weights:

(9) wk,n=1[(H(n)∗H(n))−1]ik,nik,n, k=1,2,…,K, n=1,2,…,N.

**Figure 1:**
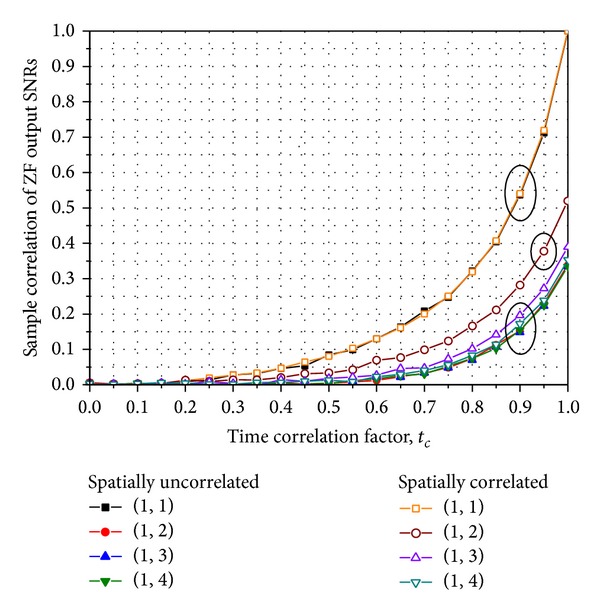
Sample correlations of ZF output SNRs for an E-SMC in Rayleigh MIMO channels: (·, ·) denotes the indices of transmit antennas in the first and the second time slots, respectively, and the correlation matrices in (41) are used.

Using (7) and (9), the MRC output SNR for the *k*th symbol is then given as

(10) $$\begin{array}{c} \gamma_{k}^{\text{mrc}}=\overset{N_{k}}{\underset{j=1}{\sum }}\gamma_{k,t_{j}^{\left(k\right)}},\;k=1,2,\ldots ,K, \end{array}$$

where *t*_*j*_^(*k*)^, *j* = 1,2,…, *N*_*k*_, denote the indices of the time slots assigned to transmit the *k*th symbol and *N*_*k*_ is the number of time slots used to transmit the *k*th symbol in the E-SMC. Since the jointly statistical analysis of *γ*_*k*,*t*_*j*_^(*k*)^_ for *j* = 1,2,…, *N*_*k*_ may be difficult in flat Rayleigh fading channels that are changing between time slots, we assume that *γ*_*k*,*t*_*j*_^(*k*)^_'s are independent. To justify the independent assumption, we plot Figure 1 that shows the sample correlations of ZF output SNRs with respect to the time correlation factor in Rayleigh fading channels. It is observed that the sample correlations for all the cases are below 0.05 and 0.22 when *t*_*c*_ ≤ 0.4 and 0.7, respectively. It means that for low time correlation, the correlation of ZF output SNRs is close to zero, and hence the second equality in (11) can be approximately yielded. We can then obtain the MGF approximation of *γ*_*k*_^mrc^ in flat Rayleigh fading channels that vary from one to another time slot. As a result, the MGF of *γ*_*k*_^mrc^ is expressed as the following equation:

(11) Mγkmrc(s)=Eγkmrc[exp⁡(−sγkmrc)]=∏j=1Nk[∫0∞exp⁡(−sαk,tj(k))fγk,tj(k)(αk,tj(k))dαk,tj(k)]=∏j=1Nk11+sρck,tj(k),

where E_*X*_[·] represents the expectation operation with respect to *X*.

Without loss of generality, we assume that *c*_*k*,*t*_1_^(*k*)^_ ≥ ⋯≥*c*_*k*,*t*_*N*_*k*__^(*k*)^_ > 0 and there exist *D*_*k*_ distinct values in {*c*_*k*,*t*_*j*_^(*k*)^_} such that

(12) ck,t1(k)=⋯=ck,tu1(k)=λ1,k,ck,tu1+1(k)=⋯=ck,tu1+u2(k)=λ2,k, ⋮ck,tu1+⋯+uDk−1+1(k)=⋯=ck,tu1+⋯+uDk(k)=λDk,k,

where *u*_1_ + *u*_2_ + ⋯+*u*_*D*_*k*__ = *N*_*k*_. Then, the MGF of *γ*_*k*_^mrc^ can be rewritten as

(13) $$\begin{array}{c} M_{\gamma_{k}^{\text{mrc}}}\left(s\right)=\frac{1}{\prod_{q=1}^{D_{k}}{\left(\rho \lambda_{q,k}\right)}^{u_{q}}}\overset{D_{k}}{\underset{q=1}{\prod }}{\left(s+\frac{1}{\rho \lambda_{q,k}}\right)}^{-u_{q}}. \end{array}$$

Using the partial fraction expansion of the MGF, it can be expressed as [14]

(14) $$\begin{array}{c} M_{\gamma_{k}^{\text{mrc}}}\left(s\right)=\frac{1}{\prod_{q=1}^{D_{k}}{\left(\rho \lambda_{q,k}\right)}^{u_{q}}}\overset{D_{k}}{\underset{q=1}{\sum }}\;‍\overset{u_{q}}{\underset{l=1}{\sum }}\mu_{q,l,k}{\left(s+\frac{1}{\rho \lambda_{q,k}}\right)}^{-l}, \end{array}$$

where

(15) μq,l,k=(−1)uq−l∑Ψ∏j=1,j≠qDk(uj−1+mjmj)(1ρλj,k−1ρλq,k)−(uj+mj),

and Ψ denotes the set of nonnegative integers {*m*_1_,…, *m*_*q*−1_, *m*_*q*+1_,…, *m*_*D*_*k*__} such that

(16) $$\begin{array}{c} m_{1}+\cdots +m_{q-1}+m_{q+1}+\cdots +m_{D_{k}}=u_{q}-l. \end{array}$$

### 3.3. SER of *M*-ary QAM for E-SMCs with ZF and MRC

Using a MGF-based approach, we write the average SER of symbol *k* in an E-SMC with *M*-ary QAM as in [15]

(17) Pe,kQAM=4π(1−1M)∫0π/2Mγkmrc(gQAMsin2ϕ)dϕ−4π(1−1M)2×∫0π/4Mγkmrc(gQAMsin2ϕ)dϕ,

where *g*_QAM_ = 3/{2(*M* − 1)}. Substituting (14) in (17) and using (5A.4a) and (5A.21) in [15], we can obtain that

(18) $$\begin{array}{c} P_{e,k}^{\text{QAM}}=\left(2-\frac{2}{\sqrt{M}}\right)J_{k,1}\left(g_{\text{QAM}}\right)-{\left(2-\frac{2}{\sqrt{M}}\right)}^{2}J_{k,2}\left(g_{\text{QAM}}\right), \end{array}$$

where

(19) Jk,1(gQAM)  =1Ω∑q=1Dk ∑l=1uqμq,l,k(ρλq,k)l   ×[1−ξq,k∑i=0l−1(2ii)(1−(ξq,k)24)i],Jk,2(gQAM)=1Ω∑q=1Dk ∑l=1uqμq,l,k(ρλq,k)l      ×[14−ξq,kπ        ×{(π2−tan−1ξq,k)         ×∑i=0l−1(2ii)×(4+4ρλq,kgQAM)−i         −sin(tan−1ξq,k)         ×∑i=1l−1 ∑j=1iTji×(1+ρλq,kgQAM)−i             ×{cos⁡(tan−1ξq,k)}2(i−j)+1}],

where

(20) $$\begin{array}{c} \Omega =\overset{D_{k}}{\underset{q=1}{\prod }}{\left(\rho \lambda_{q,k}\right)}^{u_{q}},\;\;\xi_{q,k}=\sqrt{\frac{\rho \lambda_{q,k}g_{\text{QAM}}}{1+\rho \lambda_{q,k}g_{\text{QAM}}}},\\ T_{ji}=\frac{\left(\begin{array}{c} 2i\\ i \end{array}\right)}{\left(\begin{array}{c} 2\left(i-j\right)\\ i-j \end{array}\right)4^{j}\left[2\left(i-j\right)+1\right]}. \end{array}$$

Therefore, the overall average SER of E-SMCs with ZF and MRC for *M*-ary QAM in transmit-correlated Rayleigh MIMO channels is given by

(21) $$\begin{array}{c} P_{e}^{\text{QAM}}=\frac{1}{K}\overset{K}{\underset{k=1}{\sum }}P_{e,k}^{\text{QAM}}. \end{array}$$

### 3.4. SER of *M*-ary PSK for E-SMCs with ZF and MRC

The average SER of the *k*th symbol in an E-SMC with *M*-ary PSK is given as in [15]

(22) $$\begin{array}{c} P_{e,k}^{\text{PSK}}=\frac{1}{\pi }\overset{(M-1)\pi /M}{\underset{0}{\int }}M_{\gamma_{k}^{\text{mrc}}}\left(\frac{g_{\text{PSK}}}{\text{si}{\text{n}}^{2}\phi }\right)d\phi , \end{array}$$

where *g*_PSK_ = sin^2^(*π*/*M*). Inserting (14) into (22) and using (5A.17) in [15], it can be expressed as

(23) Pe,kPSK=1Ω∑q=1Dk ∑l=1uqμq,l,k(ρλq,k)l      ×[M−1M−ζq,kπ        ×{(π2+tan−1θq,k)          ×∑i=0l−1(2ii)×(4+4ρλq,kgPSK)−i          +sin⁡(tan−1θq,k)          ×∑i=1l−1 ‍∑j=1iTji×(1+ρλq,kgPSK)−i              ×{cos⁡(tan−1θq,k)}2(i−j)+1}],

where

(24) $$\begin{array}{c} \zeta_{q,k}=\sqrt{\frac{\rho \lambda_{q,k}g_{\text{PSK}}}{1+\rho \lambda_{q,k}g_{\text{PSK}}}},\;\;\theta_{q,k}=\zeta_{q,k}\text{cot}\frac{\pi }{M}. \end{array}$$

Hence, for *M*-ary PSK, the overall average SER of E-SMCs with ZF and MRC in transmit-correlated Rayleigh MIMO channels is obtained by

(25) $$\begin{array}{c} P_{e}^{\text{PSK}}=\frac{1}{K}\overset{K}{\underset{k=1}{\sum }}P_{e,k}^{\text{PSK}}. \end{array}$$

## 4. Optimization of the Slot Length in E-SMCs

We assume that a symbol block to be encoded with the E-SMC consists of *K* complex symbols each of which is from the respective different data stream that requires *ε*_*k*_ (*k* = 1,2,…, *K*) as a target SER. Our goal is to find a minimum-length E-SMC that satisfies the SER requirement for each data stream. For symbol *k*, let

(26) $$\begin{array}{c} N_{k}^{*}=\min\left\{N_{k}\in Z^{+}\text{subject}\;\text{to}\;P_{e,k}\leq \varepsilon_{k}\right\}, \end{array}$$

where *Z* ^+^ denotes the set of nonnegative integers and *P*_*e*,*k*_ is the SER of data stream *k* obtained by either (18) or (23). It is noted that *N*_k_* can be easily found since *P*_*e*,*k*_ is a monotone decreasing function of *N*_*k*_. We assume, without any loss of generality, that *N*_1_* ≥ *N*_2_* ≥ ⋯≥*N*_K_*.

### 4.1. The Minimum Slot Length for a Single Symbol Block

When we have a single *K*-symbol block, an optimization problem to minimize the block length *N* with the required SER is

(27)  min⁡N∈Z+ N

(28)  s.t.  N1∗+N2∗+⋯+NK∗≤Nnt,

(29)     N1∗≤N.

Conditions (a) and (c) for the proposed E-SMC in Section 2.1 are satisfied when either only one symbol or nothing is transmitted at a transmit antenna in a time slot. Constraint (28) indicates that the size of a code block should be greater than or equal to that needed to transmit *K* symbols while achieving the required SERs, respectively. Constraint (29) implies that the block length cannot be less than the largest number among the required time slots for respective data streams, which is a necessary condition to maintain property (b) of the E-SMC.

From (28) and (29), an optimal block length N* is given by

(30) $$\begin{array}{c} N^{*}=\max\left\{N_{1}^{*},\left\lceil \frac{N_{1}^{*}+N_{2}^{*}+\cdots +N_{K}^{*}}{n_{t}}\right\rceil \right\}. \end{array}$$

When N* is obtained, the following simple symbol allocation method (SSM) provides an E-SMC that satisfies conditions (a)–(c) in Section 2.1. Let *a*_*ij*_^(*k*)^ denote the (*i*, *j*)th element of code matrix **A**_*k*_ for symbol *x*_*k*_ and recall *N*_1_* ≥ *N*_2_* ≥ ⋯≥*N*_K_*.

**Step 1 .**

Initialize all *a*_*ij*_^(*k*)^'s to be zero, and let *k* = 1, *L* = *N*_k_*, *i* = 1, and *j* = 1.

**Step 2 .**

If *L* = 0, then go to Step 3. Otherwise, let *a*_*ij*_^(*k*)^ = 1, *L* = *L* − 1, and *j* = *j* + 1; if *j* > N*, then let *j* = 1, *i* = *i* + 1, and repeat Step 2.

**Step 3 .**

If *k* = *K*, then stop the symbol allocation procedure since the code matrix is already obtained. Otherwise, let *k* = *k* + 1 and *L* = *N*_k_*, and go to Step 2.

**Lemma 1 .**

The above SSM always finds {**A**_*k*_} that satisfies conditions (a)–(c) in Section 2.1, the column length of which is equal to N*.

**Proof**

For mathematical induction, suppose that *N*_1_* + *N*_2_* + ⋯+*N*_k_* 1's are successfully allocated, with SSM, to the (*i*, *j*)th element in **A**_*k*_. Then, remaining *N*_*k*+1_* + *N*_*k*+2_* + ⋯+*N*_K_* 1's can be allocated until the (*n*_*t*_, N*)th element in **A**_*k*_ is considered since

(31) Nk+1∗+Nk+2∗+⋯+NK∗≤ntN∗−(N1∗+N2∗+⋯+Nk∗)=ntN∗−(i−1)N∗−j=(nt−i)N∗+(N∗−j).

Furthermore, *N*_*k*+1_* 1's allocated at **A**_*k*+1_ do not violate conditions (a)–(c) since *N*_*k*+1_* ≤ *N*_1_* ≤ N*. By (30), *N*_1_* 1's for symbol 1 are always allocated at the first row of **A**_1_, which completes the proof by mathematical induction.

For example, the following code blocks **X**_3_ and **X**_4_ are obtained by SSM: **X**_3_ for *n*_*t*_ = 4, *K* = 6, *N*_1_* = 4, *N*_2_* = 4, *N*_3_* = 4, *N*_4_* = 2, *N*_5_* = 2, *N*_6_* = 2, and hence N* = 5; **X**_4_ for *n*_*t*_ = 4, *K* = 5, *N*_1_* = 3, *N*_2_* = 3, *N*_3_* = 2, *N*_4_* = 2, *N*_5_* = 2, and hence N* = 3:

(32) X3=[x1,1x1,2x1,3x1,4x2,5x2,1x2,2x2,3x3,4x3,5x3,1x3,2x4,3x4,4x5,5x5,1x6,2x6,3],X4=[x1,1x1,2x1,3x2,1x2,2x2,3x3,1x3,2x4,3x4,1x5,2x5,3].

It is noted that (4,4)th and (4,5)th elements of **X**_3_ are unused, which implies that constraint (28) holds in a strict inequality. This unusage wastes the radio resource but could be avoided by allocating additional symbols. The following section addresses this issue further.

### 4.2. Removing Unused Code Positions in the Transmission Matrix

Once the unused position in the code matrix is removed, a maximum code rate is achieved. Thus, a problem of removing the unused optimally can be formulated as

(33)  min⁡N,c∈Z+ N

(34)  s.t.  c{N1∗+N2∗+⋯+NK∗}=Nnt,

(35)     N1∗≤N,

where *N*_1_* ≥ *N*_2_* ≥ ⋯≥*N*_K_* and the equality in (34) ensures that there is no void element in a code matrix, which makes the code rate maximized. It is noted that if the above problem has an optimal c*, then a c*K-symbol block is transmitted with a code matrix. Moreover, if c* > 1, the resulting code matrix induces additional delay compared with the code obtained in Section 4.1.

Since (34) holds in equality in order to get rid of the unused position in the transmission code matrix,

(36) $$\begin{array}{c} N^{*}=\underset{N\in Z^{+},N\geq N_{1}^{*}}{\min}\left\{N=\frac{c\left\{N_{1}^{*}+N_{2}^{*}+\cdots +N_{K}^{*}\right\}}{n_{t}},c\in Z^{+}\right\}. \end{array}$$

Let (*N*_1_* + *N*_2_* + ⋯+*N*_K_*)/*n*_*t*_ = *d* + (*e*/*n*_*t*_)  (*d*, *e* ∈ *Z* ^+^and  *e* < *n*_*t*_). If *e* = 0, N* and c* should be the smallest nonnegative integers that satisfy N* = c*d ≥ *N*_1_*. In the other case of *e* ≠ 0, let *e*/*n*_*t*_ = *e*′/*n*_*t*_′ where the greatest common denominator of *e*′ and *n*_*t*_′ is 1. Then, (34) becomes *N* = *c*{*d* + (*e*′/*n*_*t*_′)} = *ln*_*t*_′*d* + *le*′, where *c* = *ln*_*t*_′. Let l* be the smallest nonnegative integer such that l*(*n*_*t*_′*d* + *e*′) ≥ *N*_1_*; then, c* = l*n_*t*_′ and N* = l*(*n*_*t*_′*d* + *e*′).

After achieving c*, SSM described in the previous section also can be applied for getting the code matrices if c*K is used instead of *K*. For example, the previous code block **X**_3_ is now extended to

(37) X5=[x1,1x1,2x1,3x1,4x2,5x2,6x2,7x2,8x3,9x3,1x3,2x3,3x4,4x4,5x4,6x4,7x5,8x5,9x5,1x5,2x6,3x6,4x6,5x6,6x7,7x7,8x8,9x8,1x9,2x9,3x10,4x10,5x11,6x11,7x12,8x12,9],

where c* = 2 and N* = 9.

## 5. Simulation and Analytic Results

In the simulation, we consider spatially transmit-correlated MIMO channels when *n*_*t*_ = *n*_*r*_ = 4, and the transmit correlation matrix is assumed as

(38) $$\begin{array}{c} R_{t}=\left[\begin{array}{cccc} \beta_{11}^{t} & \beta_{12}^{t} & \beta_{13}^{t} & \beta_{14}^{t}\\ \beta_{21}^{t} & \beta_{22}^{t} & \beta_{23}^{t} & \beta_{24}^{t}\\ \beta_{31}^{t} & \beta_{32}^{t} & \beta_{33}^{t} & \beta_{34}^{t}\\ \beta_{41}^{t} & \beta_{42}^{t} & \beta_{43}^{t} & \beta_{44}^{t} \end{array}\right], \end{array}$$

where *β*_*ij*_^*t*^ denotes the correlation coefficient between the *i*th and the *j*th transmit antennas. In order to obtain the correlations between two antennas, we use the following approximation [16]:

(39) $$\begin{array}{c} \beta \left(d\right)\approx \exp\left(-23\Lambda^{2}d^{2}\right), \end{array}$$

where *d* represents the distance in wavelengths between two antennas and Λ denotes the angular spread. Note that Λ is defined for any distribution of power in the azimuth plain, and values close to 0.0 denote completely directional scenarios whereas those at 1.0 represent more uniform spreading of energies in space. Based on this approximation, the correlation matrix in (38) is simplified for a linear array at the transmitter with equidistant antenna spacing *d*_*t*_ as the following Toeplitz structure correlation matrix [17]:

(40) $$\begin{array}{c} R_{t}=\left[\begin{array}{cccc} 1 & \beta \left(d_{t}\right) & \beta \left(2d_{t}\right) & \beta \left(3d_{t}\right)\\ \beta \left(d_{t}\right) & 1 & \beta \left(d_{t}\right) & \beta \left(2d_{t}\right)\\ \beta \left(2d_{t}\right) & \beta \left(d_{t}\right) & 1 & \beta \left(d_{t}\right)\\ \beta \left(3d_{t}\right) & \beta \left(2d_{t}\right) & \beta \left(d_{t}\right) & 1 \end{array}\right]. \end{array}$$

From (40), the correlation matrices for uncorrelated and correlated MIMO channels considered in this section are, respectively, given by

(41) $$\begin{array}{c} R_{t}^{\text{unc}}=\left[\begin{array}{cccc} 1 & 0 & 0 & 0\\ 0 & 1 & 0 & 0\\ 0 & 0 & 1 & 0\\ 0 & 0 & 0 & 1 \end{array}\right],\\ R_{t}^{\text{cor}}=\left[\begin{array}{cccc} 1 & 0.596 & 0.126 & 0.009\\ 0.596 & 1 & 0.596 & 0.126\\ 0.126 & 0.596 & 1 & 0.596\\ 0.009 & 0.126 & 0.596 & 1 \end{array}\right], \end{array}$$

where *d*_*t*_ = 1.5 and Λ = 0.1 in the case of correlated one.

**Figure 2:**
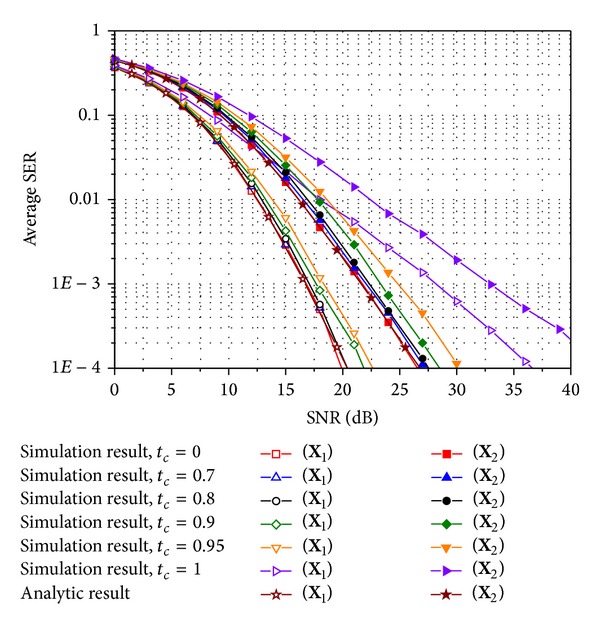
Average SER of E-SMCs with ZF and MRC for QPSK in spatially uncorrelated Rayleigh MIMO channels.

**Figure 3:**
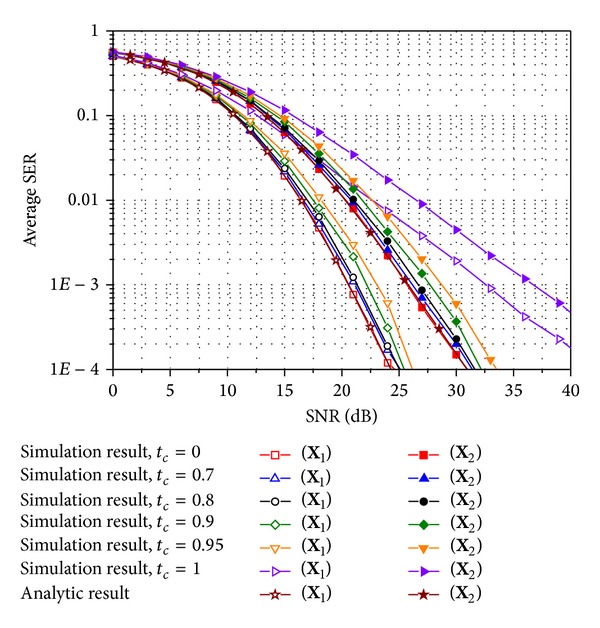
Average SER of E-SMCs with ZF and MRC for QPSK over a spatially correlated Rayleigh MIMO channel with *d*_*t*_ = 1.5 and Λ = 0.1.

**Figure 4:**
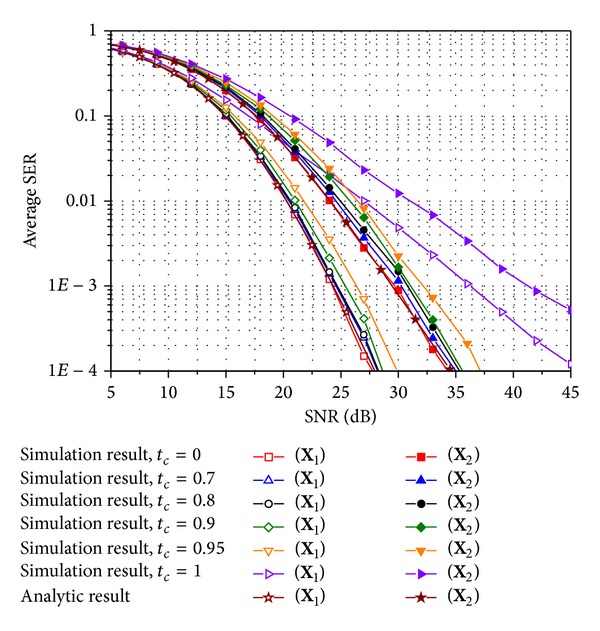
Average SER for E-SMCs with ZF and MRC with 16-ary QAM in spatially uncorrelated Rayleigh MIMO channels.

**Figure 5:**
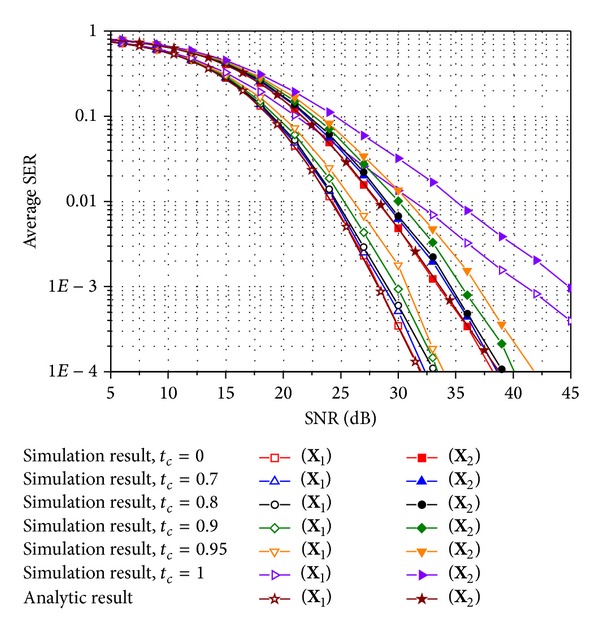
Average SER for E-SMCs with ZF and MRC with 16-ary QAM over a spatially correlated Rayleigh MIMO channel with *d*_*t*_ = 1.5 and Λ = 0.1.

To obtain the numerical results, we test **X**_1_ and **X**_2_ in (3) as E-SMCs. Figures 2–5 compare the average SER performance of the two E-SMCs with ZF and MRC at a receiver. Both simulation and analytic results are plotted for Rayleigh MIMO channels. Especially, the simulated curves are obtained using time-varying channels with various time correlation factors defined in (6). In particular, Figures 2 and 3 show the average SER for QPSK in uncorrelated and correlated Rayleigh channels with **R**_*t*_^unc^ and **R**_*t*_^cor^, respectively, and Figures 4 and 5 also exhibit the average SER for the same scenario used in Figures 2 and 3 except that 16-ary QAM is investigated instead of QPSK. In comparing the analytic SER curve obtained using the MGF approximation with the simulated curves, it is clear that the analytic curve is perfectly matched with the simulated one with *t*_*c*_ = 0 irrespective of the modulation schemes and the spatial correlations. The gap between the simulated result for a time correlation factor of zero and that for a time correlation factor less than 0.8 does not exceed 1 dB. Hence, approximate SER expressions (18) and (23) for *M*-ary QAM and PSK, respectively, can be used to study SER performance for E-SMCs with ZF and MRC over time-varying Rayleigh MIMO channels with rather a low correlation between time slots. Those four figures also illustrate that **X**_1_ with *R*_*c*_ = 4/3 provides better SER performance than **X**_2_ with *R*_*c*_ = 2 from a tradeoff between the coding rate in (2) and SER performance. In addition, SER performance becomes better as the time correlation factor decreases since the time diversity gain increases as the channels between time slots vary more independently. An increase in the correlation between transmit antennas induces the degradation of SER performance, while it does not affect the approximation gap between **X**_1_ and **X**_2_ as well as the simulation results with various time correlation factors.

**Figure 6:**
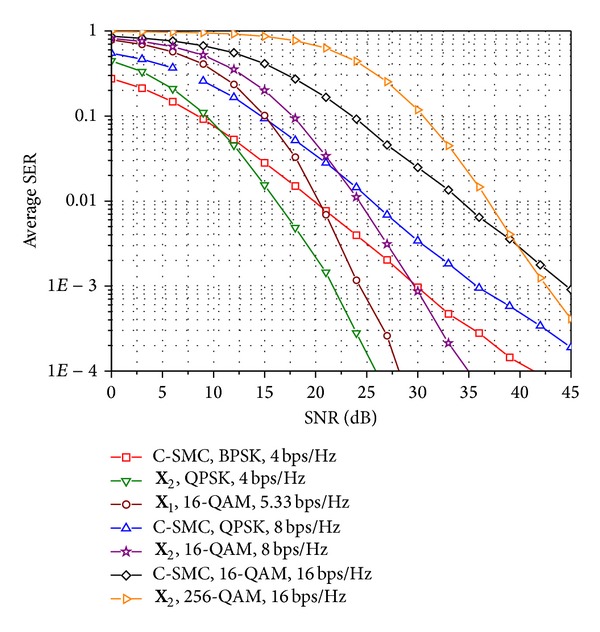
Comparison of SER performance for the conventional SMC and the E-SMC in spatially uncorrelated Rayleigh MIMO channels when *t*_*c*_ = 0 and *n*_*t*_ = *n*_*r*_ = 4.

**Figure 7:**
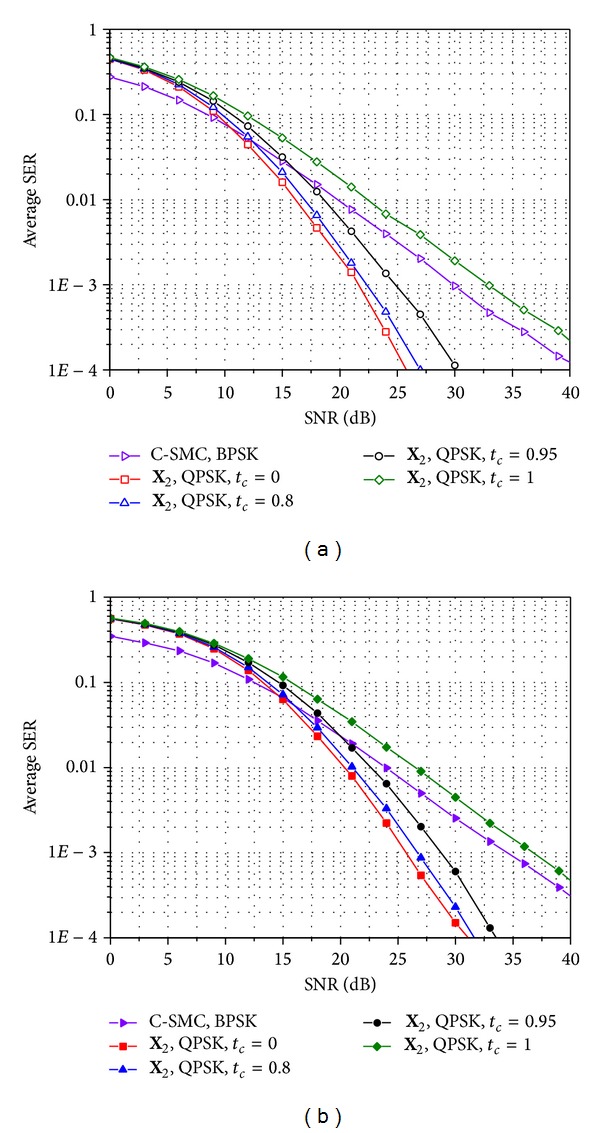
Comparison of SER performance for the conventional SMC and the E-SMC with *R*_*s*_ = 4 bps/Hz when *n*_*t*_ = *n*_*r*_ = 4: (a) spatially uncorrelated Rayleigh MIMO channels and (b) spatially correlated Rayleigh MIMO channels with *d*_*t*_ = 1.5 and Λ = 0.1.

**Figure 8:**
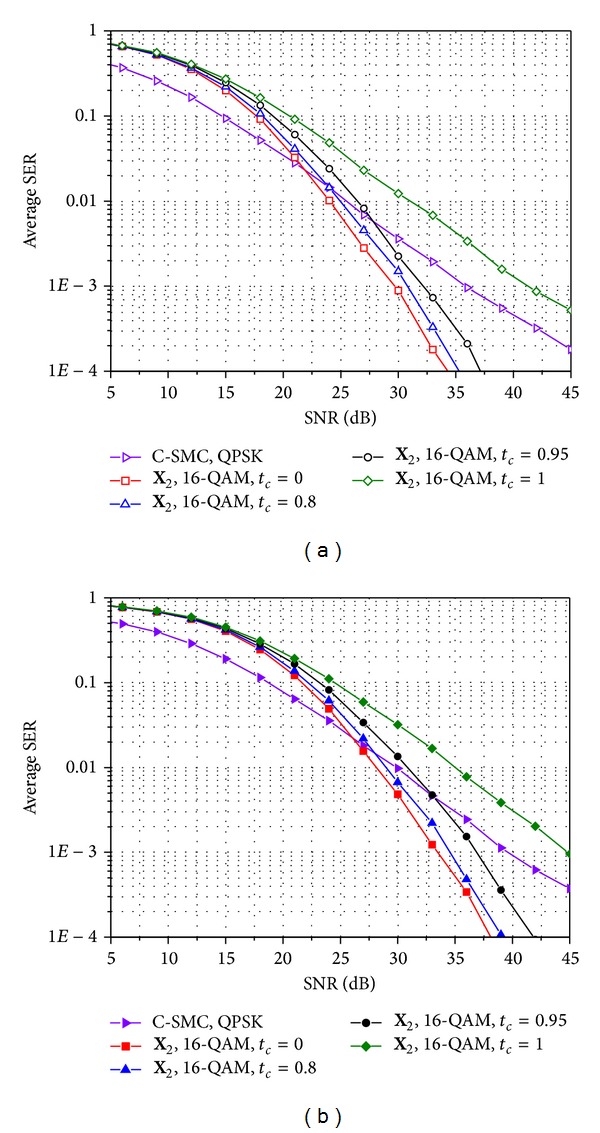
Comparison of SER performance for the conventional SMC and the E-SMC with *R*_*s*_ = 8 bps/Hz for *n*_*t*_ = *n*_*r*_ = 4: (a) spatially uncorrelated Rayleigh MIMO channels and (b) spatially correlated Rayleigh MIMO channels with *d*_*t*_ = 1.5 and Λ = 0.1.

Figures 6–8 compare the SER performances of the conventional SMC, indicated as “C-SMC” in the figures, and E-SMCs for various transmission rates, *R*_*s*_, that are defined as

(42) $$\begin{array}{c} R_{s}=R_{c}\log_{2}M, \end{array}$$

where the unit of *R*_*s*_ is bps/Hz. In Figure 6, it is demonstrated that the E-SMC performs better than the conventional SMC at high SNR and takes lower modulation order for an equal transmission rate when the channels between time slots are independent. The SER curves for E-SMCs in Figure 6 give lower bounds since the time diversity gain is maximally achieved when *t*_*c*_ = 0. Through the comparison in Figure 6, we can see the maximum achievable gain of E-SMCs. More specifically, in Figures 7 and 8, we compare the SER performances of the conventional SMC and E-SMCs in time-varying and correlated Rayleigh MIMO channels when rates are given as *R*_*s*_ = 4 bps/Hz and 8 bps/Hz, respectively. In Figures 7 and 8, it is seen that the E-SMC offers better SER performance than the conventional SMC over high SNRs even for high time correlation factors. However, SER performance for E-SMCs becomes worse when the channel does not change over time slots (i.e., *t*_*c*_ = 1) since the gap between modulation gains of BPSK and QPSK or QPSK and 16-ary QAM is larger than 3 dB that is achieved by using MRC for the same two received signals. In comparison between Figures 7(a) and 7(b) as well as Figures 8(a) and 8(b), the spatial correlation between transmit antennas does not affect the performance gap between the conventional SMC and the E-SMC.

**Figure 9:**
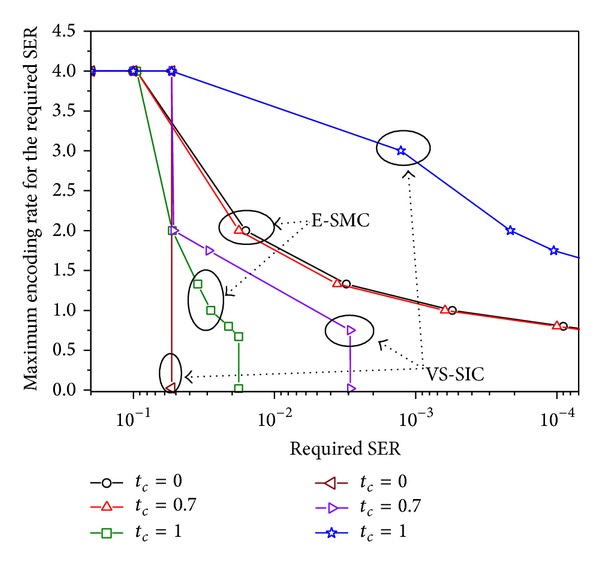
Comparison of maximum encoding rates achieved by the E-SMC and the VS-SIC in time-varying and spatially uncorrelated Rayleigh fading channels when *ρ* = 15 dB and QPSK is used.

Figure 9 compares maximum encoding rates achieved by E-SMCs and vr-STBCs with successive interference cancellation (VS-SIC) [8] in time-varying and spatially uncorrelated Rayleigh fading channels when *ρ* = 15 dB and QPSK is used. The maximum encoding rate to meet the required SER is obtained by using the sets of available encoding rates {4,2, 4/3,1, 4/5,2/3} and {4,3, 2,7/4,3/4} for the E-SMC and the VS-SIC, respectively. In this figure, we draw a linear line between two adjacent results for the sake of illustration. In Figure 9, it is indicated that in time-varying channels with low time correlation the E-SMC can achieve time diversity, while the VS-SIC achieves no spatial diversity. Also, the VS-SIC is more sensitive to the time correlation factor than the E-SMC.

**Figure 10:**
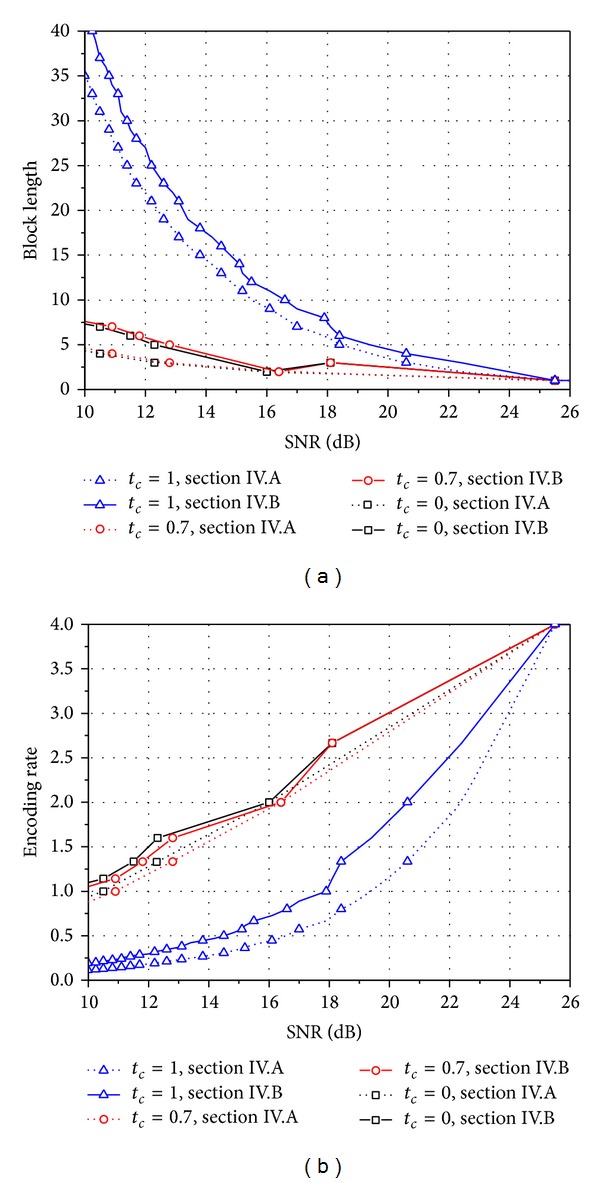
(a) Optimal block length and (b) its encoding rate for *ε*_1_ = *ε*_2_ = 0.01 and *ε*_3_ = *ε*_4_ = 0.05 in spatially uncorrelated Rayleigh MIMO channels when *n*_*t*_ = *n*_*r*_ = 4 and QPSK is used.

Figure 10 shows an optimal block length and its encoding rate for given target SER *ε*_1_ = *ε*_2_ = 0.01 and *ε*_3_ = *ε*_4_ = 0.05 in spatially uncorrelated Rayleigh MIMO channels when *n*_*t*_ = *n*_*r*_ = 4 and QPSK is used. In Figure 10(a), we employ the SSM to find *N*_k_* for symbol *k* in (26) and then the optimal block lengths for various SNRs are obtained by using (30) in Section 4.1 and (36) in Section 4.2. In Figure 10(b), the encoding rates are then obtained from the optimal block lengths in Figure 10(a). Using (36) in Section 4.2 offers a higher encoding rate than using (30) in Section 4.1 but induces an increase in the block length.

## 6. Conclusion

In this paper, we have proposed E-SMCs, which incorporate the symbol repetition over time. The E-SMC leads to a tradeoff between encoding rate and time diversity. For MIMO systems using E-SMCs, the received signals are decoded by ZF and MRC in this paper. SER equations of *M*-ary QAM and PSK are then derived by using a MGF approximation for SNR under the independence assumption on the ZF output SNRs. With numerical investigation, simulation and analytical results are compared for time-varying and spatially correlated Rayleigh fading channels. Numerical results show that the analytical SER given by the approximation is exactly matched with the simulated one when there is no correlation of channels between time slots, and the gap between the SER performance for zero time correlation and that for low time correlation below 0.8 is less than 1 dB, regardless of the spatial correlation. The results also show that, at high SNR and low time correlation, the E-SMCs outperform the conventional SMC and the vr-STBCs in terms of SER and data rate, respectively.
